# Novel Electrochemical Sensors Based on L-Proline Assisted LDH for H_2_O_2_ Determination in Healthy and Diabetic Urine

**DOI:** 10.3390/s22197159

**Published:** 2022-09-21

**Authors:** Mauro Tomassetti, Riccardo Pezzilli, Giuseppe Prestopino, Corrado Di Natale, Pier Gianni Medaglia

**Affiliations:** 1 Department of Electronic Engineering, University of Rome ‘‘Tor Vergata’’, Viale del Politecnico 1, 00133 Rome, Italy; 2 Department of Chemistry, University of Rome “La Sapienza”, P.le A. Moro 5, 00185 Rome, Italy; 3 Department of Industrial Engineering, University of Rome ‘‘Tor Vergata’’, Viale del Politecnico 1, 00133 Rome, Italy

**Keywords:** proline-assisted LDH amperometric sensor, voltammetry, amperometry, H_2_O_2_ determination in healthy and diabetic human urine

## Abstract

In this paper, a novel non-enzymatic modified glassy carbon (GC) sensor, of the (GC-Ag_paste_)-catalytic proline-assisted LDH type, for H_2_O_2_ determination was fabricated, studied, characterized and employed to determine the hydrogen peroxide content in healthy and diabetic human urine. LDH (whose composition can be schematized as [Zn^II^Al^III^ (OH)_2_]^+^ NO_3_^−^·nH_2_O) is glued to glassy carbon by means of silver paste, while proline, which increases the catalytic properties of LDH, is used free in solution in the phosphate buffer. A voltametric survey was first conducted to ascertain the positive effect induced by the presence of proline, i.e., the increase of sensor sensitivity. Then a deep study of the new three-electrode amperometric proline-assisted LDH sensor, whose working electrode was of the same type as the one used to perform the cyclic voltammetry, was carried out, working at first in static air, then in a nitrogen atmosphere. Possible interferences from various substances, both oxidants and antioxidants, were also investigated. Lastly, the new amperometric sensor was successfully used to determine the H_2_O_2_ level in human urine from both healthy and diabetic subjects. The effect of proline in enhancing the properties of the sensor system was also investigated. The limit of detection (LOD) of the new catalytic sensor was of the order of 0.15 mmol L^−1^, working in air, and of 0.05 µmol L^−1^, working in nitrogen atmosphere.

## 1. Introduction

The determination of hydrogen peroxide is of considerable importance in chemistry, since H_2_O_2_ is present in numerous real matrices, such as several pharmaceutical disinfectant preparations [1], is used in the treatment of organic waste and industrial stream [2], in the agro-food industry [2] (especially in the dairy industry [3]), and it is also contained in many cosmetic products [4]. In more recent times, the importance of H_2_O_2_ has also been recognized in the biomedical field; in fact, it is believed that H_2_O_2_ plays a very important role both in cell proliferation and death and in the transduction of intracellular signals [5,6]. Therefore, a high H_2_O_2_ level in biological systems is associated with oxidative stress and neurodegenerative disorders [7], so much so that the endogenous hydrogen peroxide efflux in living cells deserves to be evaluated as a biomarker for cancer diagnostics [8]. Hence, the need to develop very sensitive methods of H_2_O_2_ determination in biological fluids such as plasma and urine and even in living cells [9] has gained increasing attention. Colorimetric [10,11], spectrophotometric [12,13,14], enzymatic [15,16], and chemiluminescent [17] methods for hydrogen peroxide determination are well known and widely used. However, electrochemical methods are often preferred, since they are generally faster, direct, and less expensive [18,19,20]. It should also be noted that in most of the above-mentioned applications, very sensitive analysis routes featuring very low detection limits are required; non-invasiveness, practicality, and possibly low cost are also desired. Among these, the electroenzymatic methods [21,22] are the most used, since they are generally selective, as well as sensitive and specific. However, electroenzymatic methods, like those based on enzymatic biosensors, as well as all enzymatic methods, are generally quite expensive and their response is often not very stable, especially if used for long periods without the enzyme being properly stored. They also require a good immobilization of the enzyme on a special support, which complicates practical use. For these reasons, the development of non-enzymatic electrocatalytic platforms for the determination of H_2_O_2_ has been very dynamic in recent years, mainly based on nanomaterials of noble metals such as silver, gold, or platinum [23,24,25,26,27,28]. Electrocatalytic sensors based on metals such as copper, iron, manganese, or their oxides [29], or on silver oxides [23] have also been developed. Recent research efforts to obtain new types of electrocatalytic sensors, responsive, for example, to H_2_O_2_, have led to the introduction of atomically thick metal multicomponent active supports. Among them, the so-called layered double hydroxides (LDHs), a class of inorganic materials with layered structure, have proven to be very useful and suitable for the development of electrocatalytic sensors [30,31,32,33]. In this regard, we recently published [34] the development of a glassy carbon (GC-Ag_paste_)-LDH-catalase enzyme biosensor and a non-enzymatic amperometric glassy carbon sensor of the same type, for the determination of hydrogen peroxide, both based on layered double hydroxide of the [Zn^II^Al^III^ (OH)_2_]^+^ NO_3_^−^ · nH_2_O type. The former, being a biosensor, naturally resulted in a greater sensitivity than the latter, in fact limits of detection (LOD) of 0.2 mmol L^−1^ and of 1.0 mmol L^−1^ were found, respectively. In a subsequent work, in order to overcome some practical issues in the measurement of real samples containing H_2_O_2_, a second type of enzymatic biosensor was also developed [35], again based on LDH and catalase, but using a Clark-type electrode rather than a GC electrochemical transducer. Despite the good results already obtained with the previously reported [34] enzymatic (GC-Ag_paste_)-LDH-catalase biosensor (linearity range 0.2–160 mmol L^−1^), the present work conversely deals with the possibility of significantly improving the performance of the more-simple (GC-Ag_paste_)-LDH non-enzymatic sensor. In fact, recent reports on non-enzymatic electrocatalytic sensors, usually based on nanocomposites, report outstanding performance in detecting hydrogen peroxide [36,37,38,39,40]. Among these, the ones that most impressed us are those sensors that used LDH [32,36,37,40] or make use of L-proline [38,39,41]. We therefore tested whether the response of our previous catalytic non-enzymatic sensor, not yet studied in depth [34], a glassy carbon-modified by LDH, the latter glued on the GC by means of silver paste, could be significantly improved by the presence of L-proline in solution. Indeed, the preliminary results have been very positive; in the present paper we studied this amperometric sensor thoroughly, also applying it to the determination of hydrogen peroxide in real matrices (human urines).

## 2. Materials and Methods

### 2.1. Materials

Zinc nitrate hexahydrate (Zn (NO_3_)_2_ · 6 H_2_O) and aluminium nitrate nonahydrate (Al (NO_3_)_3_ · 9 H_2_O), L-proline (BioUltra, 99.5%), potassium permanganate (ACS), iron (III) chloride, glucose monohydrate, L(+)-ascorbic acid, and folic acid were supplied by Sigma-Aldrich (Steinheim, Germany). Sodium hydroxide and 0.1 mol L^−1^ pH 7 phosphate buffer solution (PBS) were from Fisher Scientific (Loughborough, UK), the uric acid and potassium chloride from Fluka BioChemika (Buchs, Switzerland), and the sodium nitrite and sodium nitrate (analytical grade) from Carlo Erba (Milan, Italy). Silver paste was also used (Agar Scientific, Stansted Essex, England, UK, 60% solid silver in 4-methylpentan-2-one).

### 2.2. LDH Preparation and Characterization

In our previously published works [34,35], hydrothermal growth [42], a mild method, was used to obtain the [Zn^II^Al^III^ (OH)_2_]^+^ NO_3_^−^·nH_2_O LDH, hereinafter referred to as (Zn-Al-NO_3_, see Figure S1). This method was chosen over several other synthesis routes, for example the simple coprecipitation method [43], the sol-gel technique [44], or the fabrication of different LDH compounds through anion exchange reactions or calcination-reconstruction methods [45,46], since it was considered a “green method” [47,48]. In this work, the coprecipitation method was used for the synthesis of LDH. This choice was the most convenient for practical reasons; indeed, the hydrothermal growth method previously used [41,46] does not have a very high synthesis yield compared to the coprecipitation method [43]. For this reason, in the present work, we opted for the latter method.

For this purpose, to obtain LDH of the type (Zn-Al-NO_3_), 50 mmol L^−1^ of aluminium nitrate and 150 mmol L^−1^ of zinc nitrate were dissolved in 200 mL of distilled and deionized water and then the solution was adjusted to pH 10 with NaOH in a closed container; the latter was placed in an oven at 90 °C for 12 h. The precipitate, i.e., LDH, was centrifuged and washed repeatedly with ethanol and deionized water, then dried at 45 °C and stored at room temperature.

The structural characterization of the product obtained was carried out by X-ray diffraction (XRD). A RIGAKU Geigerflex θ–2θ Bragg–Brentano diffractometer equipped with a Cu target (λ_Cu_ Kα = 1.5418 Å), refurbished with a goniometer control system by DFP Technologies, and equipped with a Cyberstar scintillation detector was used. In Figure S2, the XRD pattern of the LDH, prepared by coprecipitation, is compared with that of the LDH previously obtained by means of the hydrothermal grown process [34]. A perfect superimposition of the two XRD spectra can be clearly observed, indicating the same layered structure of the two raw materials.

### 2.3. Sensor Preparation

The GC electrodes were modified according to the same format used in our previous work [34]. A schematic of the modified electrode is shown in Figure S3A. First, the surface head of the GC cylindrical rod (0.5 cm in diameter) was polished with sandpaper, rinsed with deionized water and ethanol, and finally dried. Then, 15 mg of (Zn-Al-NO_3_) LDH, gently homogenized, was glued on the GC electrode surface, which was previously smeared with a very thin coat of silver paste glue. Finally, the cylindrical head of the LDH-modified GC electrode was gently screwed to the end of the electrode stem.

In the Figure S3B, a schematic of the (GC-Ag_paste_)-catalytic proline-assisted LDH electrode, used in the present paper, is reported for comparison.

### 2.4. Electrochemical Apparatus and Experimental Measurements

#### 2.4.1. Cyclic Voltammetry

All cyclic voltammetric measurements (CVs) were made from −1.5 V to +1.5 V with a scan rate of 40 mV s^−1^ using a VersaSTAT3 Potentiostat (AMETEK Scientific Instruments, Princeton, NJ, USA), a glass cell (thermostated at 25 °C), and three electrodes, namely, the working electrode (i.e., the modified GC electrode), a platinum counter electrode, and a reference electrode of Ag/AgCl/Cl^−^. The three electrodes were dipped in 40 mL of 0.1 mol L^−1^, pH 7, phosphate buffer, 50 mmol L^−1^ in KCl and 20 mmol L^−1^ in L-Proline. The trends of the CV curves and the related blanks were recorded up to a final concentration of hydrogen peroxide equal to 3.34 mmol L^−1^. The voltammetric calibration curves were obtained by reading the current peak at −0.58 V vs. Ag/AgCl/Cl^−^, i.e., practically at the same potential identified by Zhao et al. [38], by adding different volumes (20–200 µL) of 10% H_2_O_2_ solution.

#### 2.4.2. Amperometric Measurements

Amperometric experiments were carried out under the same conditions as the CV measurements using the same apparatus and the same three-electrode system, but under stirring (i.e., using a magnetic stirrer and a fly). In constructing the calibration curves, the change in volume after each addition of the hydrogen peroxide solution was taken into account, even though the volume variations were minimal.

Before performing amperometric measurements, the current response of the catalytic sensor was allowed to stabilize, with the electrode dipped in buffer solution for at least 5 min under gently stirring at a constant anode–cathode voltage difference equal to −0.58 V. After that, the calibration curves were collected by adding, time by time, subsequent fixed volumes (i.e., 150–200 µL) of 3% standard H_2_O_2_ aqueous solution, and observing as the current intensity varied immediately after each addition due to the immobilized LDH which catalysed the following reaction:

(1) $$2H_{2}O_{2}\overset{\mathrm{LDH}\;\mathrm{Catalyst}}{\to }{\;O}_{2}+2H_{2}O$$

The produced oxygen was immediately reduced at the cathode of the GC-modified electrode according to Reaction (2):

(2) $$O_{2}+4H^{+}+4e^{-}\overset{}{⟶}2H_{2}O$$

causing the variation of the current in the external circuit which was recorded once the steady state was reached after each addition.

The possible interfering substances were also tested, following the same experimental format used to test the response to standard H_2_O_2_ solutions, by comparing the sensor response in the absence and in the presence of each of the tested interferents contained in solution at the same concentration as H_2_O_2_.

The same measurements were also performed under a nitrogen stream according to the same protocol explained above. In this case a special thermostated sealed cell was employed inside which a nitrogen atmosphere was maintained, instead of air, after the sample itself was deprived of dissolved oxygen by bubbling nitrogen until saturation.

The application of the standard addition method was performed, using the standard additions of hydrogen peroxide solutions, operating in matrix (healthy or diabetic urine), in practice with the same format described above.

#### 2.4.3. Real Sample Analysis

The analyses of urine samples (donated by two subjects), recognized by the health authority as healthy and suffering from diabetes, respectively, were conducted with the same amperometric methods described above. However, in this case, the calibrations were carried out “in matrix”, using the following format. The three electrodes were initially immersed in 20 mL of the usual phosphate buffer, KCl, and proline solution, under stirring. After the current was stabilized, the first current reading was taken. Then, 20 mL of the urine sample was quickly added, and, after new stabilization, the second current reading was taken. Finally, successive additions were made, time by time, of 50 µL of 0.3% H_2_O_2_ standard solution, reading the current variations after each new addition.

The H_2_O_2_ concentration in the sample was calculated both with the linear interpolation method of the standard calibration curve built in matrix [37] and with the Gran’s plot method [49,50]. Obviously, the effective concentration of H_2_O_2_ present in the urine sample was found by doubling the concentration obtained with these two methods, since in our measurements the initial concentration of the 20 mL of sample was halved in the 40 mL of the final solution analysed; also in this case, the variations in the volume of the solution after each addition of hydrogen peroxide was taken into account, although they were minimal.

#### 2.4.4. Clark Catalytic LDH Amperometric Sensor ·Preparation and Measurements·

To better clarify the effect of proline and verify where it actually positively acted on the used (GC-Ag_paste_)-catalytic proline-assisted LDH—that is, on Ag, on LDH, or both—a modified Clark-type amperometric sensor very similar to the one described in our previous work [35] was also fabricated but without using, of course, any enzyme. To this end, 15 mg of the synthesized (Zn-Al-NO_3_) LDH and 30 mg of L-proline were placed in the center of a damp dialysis membrane (D-9777, Sigma-Aldrich) and then gently homogenized after adding two drops (about 10 µL) of pH 7 and 0.1 mol L^−1^ phosphate buffer. Lastly, the dialysis membrane containing the LDH and proline was gently stretched at the end of a Clark electrode using a small rubber O-ring, so that the LDH and proline remained rinsed between the dialysis and the gas permeable membranes. The cathode was a cylindrical platinum bar (1.0 mm in diameter) biased at −0.6 V with respect to the anode. The latter was a small cylindrical tube (5.65 mm in diameter) made of Ag/AgCl/Cl^−^, concentric to the cathode and separated from it by a plastic insulator. Anode and cathode were enclosed in a stainless-steel case (12 mm in diameter) closed at one end by a gas-permeable membrane (BO5279B from YSI incorporated, Yellow Spring Instrument, Yellow Springs, OH, USA), which was stretched and fastened with a rubber O-ring. The inner tube was filled with KCl 0.05 mol L^−1^ solution. The amperometric measurements were performed in a glass cell thermostated at 25 °C, under stirring. The tip of the assembled electrode was then dipped in 20 mL of 0.1 mol L^−1^ phosphate buffer, in the glass cell, after applying a constant (Pt) cathode–anode voltage of −0.6 V. The sensor response was allowed to stabilize for about 20 min, thereby allowing the buffer solution to permeate the dialysis membrane, solubilizing the proline rinsed between the two membranes. After that, a calibration curve was built by adding 50 µL of 3% by weight hydrogen peroxide standard solution each time. A current variation in the external circuit was observed after each addition, owing to the immobilized LDH catalysed the same reaction (1) reported above. The produced oxygen, after crossing the gas-permeable membrane, was immediately reduced at the cathode of the Clark electrode, causing the prompt variation of the output current. Also in this case, the volume changes after each addition were considered when determining the calibration curves. A “blank” calibration curve was also constructed by assembling the sensor as described above, but without using the proline rinsed between the two membranes, in order to evaluate the extent of the possible catalytic action of LDH alone. The same experience was then repeated, intercalating silver paste between the two membranes and building the same calibration curves, with and without proline.

## 3. Results and Discussion

LDH compounds exhibit a certain catalytic property towards H_2_O_2_ as reported in the literature [34,35,36,37,40]; accordingly, as already mentioned in the Introduction, our research group has recently built [34] a simple catalytic sensor, working on the basis of Reaction (1) in Section 2.4.2, which made it possible to build an amperometric electrode, which was recently described by us using this kind of LDH compound: [Zn^II^Al^III^ (OH)_2_]^+^ NO_3_^−^·nH_2_O [34]. In fact, the oxygen produced by the catalytic reaction was reduced to the GC cathode, according to Reaction (2) in Section 2.4.2. 

This previously published non-enzymatic catalytic amperometric system [34], from the analytical point of view, showed a LOD (limit of detection) of about 1.0 mmol L^−1^, a linearity range between about 145 and 1195 mmol L^−1^, and a rather modest calibration sensitivity approximately equal to 0.001 mA/mmol L^−1^. However, H. Heli et al. [36] studied a three-electrode amperometric non-enzymatic system for the determination of H_2_O_2_ based on a modified working electrode of CP/MWCNTs/CoAl-LDH, where CP (carbon paste) and MWCNTs (multiwall carbon nanotubes), with a LOD of 0.005 mmol L^−1^, therefore using a sensor little more complex than our previous sensor, i.e., [two electrodes and simple (GC-Ag_paste_)-LDH, Figure S3A], but not that different, which seems however to have very good performance, superior to that of our sensor (with a LOC of the order of 0.1 mmol L^−1^). Xu et al. [40] described a gold NP (nano particle) amperometric sensor, modified using a CoMn-LDH, which showed a LOD of 0.06 µmol L^−1^. Furthermore, Asif et al. [37] studied and built three-electrode non-enzymatic sensors for the determination of H_2_O_2_, both voltammetric and amperometric, based on a GC electrode modified with Fe_3_O_4_NSs/CuAl-LDH nanohybrids (where Fe_3_O_4_NSs = iron oxide-based nanospheres). This system showed a linear range as broad as eight orders of magnitude and a LOD even of the order of nanomoles.

These reports clearly demonstrate that LDH alone exhibits considerable catalytic properties, obviously dependent on the metal ions contained in the crystal structure and on the type of transducer electrode it interacts with. However, another very important aspect, already reported in the literature [37] but very current, according to a recent article by F. Zhao et al. [38], is the effect of some substances, in particular proline, present in solution as such or electropolymerized on the transducer, which seems to greatly increase the sensitivity of electrochemical sensors (voltammetric and amperometric) of the modified GC or CP type. Among the examples proposed in the literature, the following sensors can be cited: one that describes a GC modified with electropolymerized proline [38] for the determination of H_2_O_2_, or those concerning CPs modified with ordered mesoporous carbon (OMC) and proline assisted for the determination of different oestrogens [41] or, more specifically, estriol [39]. In particular, the article published on the determination of hydrogen peroxide [38], concerning a non-enzymatic sensor of the GC-AgNPs-proline (also named “L-proline-assisted silver nanoparticles”) type was extremely interesting for our purposes; that is, the sensor based on Ag (or Ag_2_O) nanoparticles coated with proline, obtained through a process of cyclic voltammetry in the presence of proline, AgNO_3_, and KNO_3_ in solution, and so electrodeposited on a GC transducer. In fact, this modified amperometric sensor showed a linearity range, towards H_2_O_2_, between 0.0001 and 5.15 mmol L^−1^ and an LOD of about 0.05 µmol L^−1^, extremely interesting for a non-enzymatic electrocatalytic sensor aimed at the determination of hydrogen peroxide. These highly positive performances seem to be attributed both to the very extensive surface of the Ag nanoparticles and to the metal itself (silver) due to its high conductivity and excellent catalytic activity toward H_2_O_2_, therefore presenting one of the more suitable catalytic materials for H_2_O_2_ sensors’ assembly [23]. However, in the article [38], it seems that these excellent performances are largely due to the presence of proline, although its function is not yet completely clear (in this regard also see what is reported later in this article, at the end of the paragraph “Results and Discussion”). Reflecting on these important results reported in the literature, we observed that our previously built non-enzymatic sensor [34], consisting of LDH glued to a GC transducer using silver paste (which, with prolonged exposure to the buffer solution, becomes very similar to a surface of porous silver), was not very different from that of GC-AgNPs used in the article published by F. Zhao et al. [38]. We therefore hypothesized that, even in our case, using meantime a three-electrode system and above all a sensor built according to the scheme, (GC-Ag_paste_)-catalytic proline-assisted LDH, its catalytic properties towards H_2_O_2_ could be significantly increased, thanks to the presence of proline. However, these are properties that the sensor, at least in part, already possesses thanks to the presence of the LDH immobilized on it [36,37,40], although these catalytic properties seem to vary, even significantly, based on the type of LDH used. Therefore, in the present research we have experimented with three-electrode systems for the determination of hydrogen peroxide, both voltammetric and amperometric, based on non-enzymatic (GC-Ag_paste_)-catalytic proline-assisted LDH sensors. The experimental results, reported below, certainly very positive, seem to fully support the above hypothesis, especially as regards the importance exercised by proline, which seems to increase the catalytic properties of our system.

**Figure 1:**
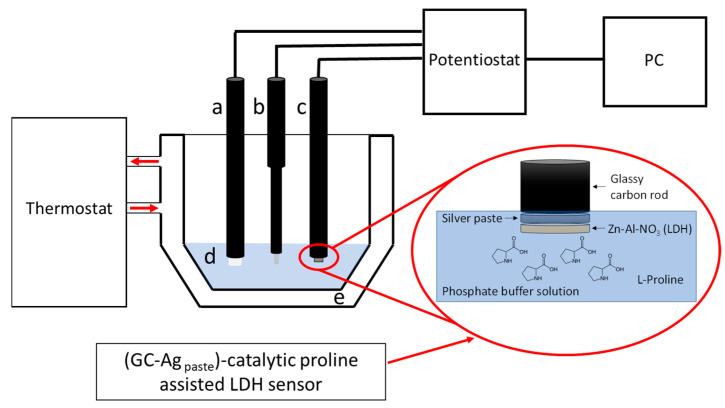
Three-electrode voltammetric system used in the present research: (**a**) counter electrode, (**b**) reference electrode, (**c**) working electrode, (**d**) proline in PBS, and (**e**) thermostated electrochemical cell. In the insert a schematic representation of the sensor (GC-Ag_paste_)-catalytic proline-assisted LDH is shown.

Inspired by such important results, and since F. Zhao et al. [38] showed that, in a voltammetric three-electrode system using electropolymerization and cyclic voltammetry between −0.8V and 1.5V in PBS, Ag nanoparticles, and proline on glassy carbon electrode, there is an increase in sensitivity towards hydrogen peroxide due to the proline coating of Ag nanoparticles, we carried out similar CV measurements in PBS containing proline using our previously built (GC-Ag_paste_)-LDH sensor [34] for the determination of H_2_O_2_ (Figure 1).

**Figure 2:**
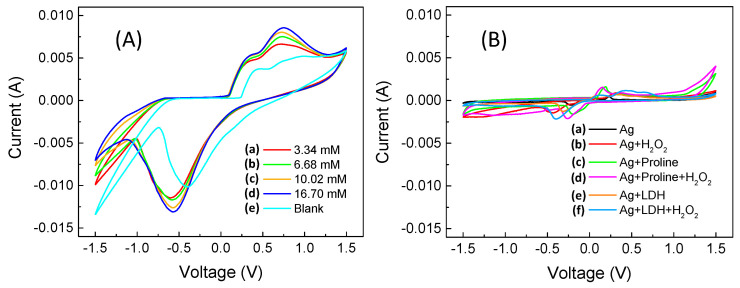
Cyclic voltammograms from −1.5 V to +1.5 V with a scan rate 40 mV s^−1^ (**A**) obtained using the sensor (GC-Ag_paste_)-catalytic proline-assisted LDH (with 20 mmol L^−1^ proline in solution), with H_2_O_2_ in phosphate buffer with final concentration of (a) 3.34, (b) 6.68, (c) 10.02, (d) 16.70 mmol L^−1^, and (e) without H_2_O_2_ in buffer solution. (**B**) “CV Blanks” obtained using (a) GC-Ag_paste_, (b) GC-Ag_paste_ in 3.34 mmol L^−1^ of H_2_O_2_, (c) GC-Ag_paste_ and proline (20 mmol L^−1^), (d) GC-Ag_paste_ and proline (20 mmol L^−1^) in 3.34 mmol L^−1^ of H_2_O_2_, (e) GC-Ag_paste_ -LDH, and (f) GC-Ag_paste_-LDH in 3.34 mmol L^−1^ of H_2_O_2_.

The measured cyclic voltammetric curves are shown in Figure 2A, while the “blank” voltammetric curves are reported in Figure 2B using the same scales from Figure 2A.

In the interval in which we performed the cyclic voltammetry (Figure 2A,B), we are not aware that there are any redox phenomena related specifically to proline (also based on what is reported in the literature). Meanwhile, as regards LDH, the peaks observed in the cathodic section of the voltammograms in both Figure 2A,B, are those relating to the formation of metal hydroxides, Zn(OH)_4_^2−^ and Zn(OH)_3_^−^, and so on [51,52], see Tomassetti et al. [34], which can be recognized on the basis of the not very intense hump at around 0.4 V. In addition, in Figure 2B we observe the oxidation processes of silver around 0.2 V (reported in the literature [38] at around 0.25 V) and silver reduction (highlighted in the literature [38] at around −0.2 V, but which, in our voltammograms, we observe at −0.35 V). As regards the role of both proline and LDH, it is only that of increasing the intensity of the redox peaks, which actually has greater importance to this research, i.e., the oxygen reduction peak at −0.57 V and the oxidation peak of hydrogen peroxide at +0.7 V (see Figure 2A).

**Figure 3:**
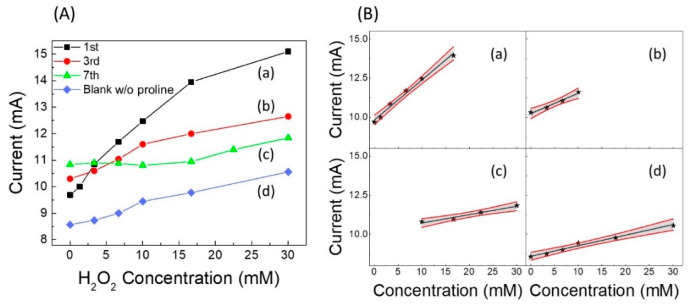
(**A**): (a–c) calibration curves vs. H_2_O_2_ concentration as a function of lifetime (about a week), reading the current peak at −0.58 V, and (d) “blank” curve without proline. (**B**) Calibration straight-lines (black) and confidence limits (red) at the (a) 1st day, (b) 3rd day, (c) 7th day, and (d) blank without proline. Each point (i.e., star symbol) is the mean of at least three determinations.

After having optimized the quantity of proline to be used, which was found to be 20 mmol L^−1^ (see Figure S4), we built a calibration curve vs. the increasing concentration of H_2_O_2_ [curve (a) in Figure 3A] by means of cyclic voltammetry carried out in presence of a fixed concentration of proline (20 mmol L^−1^) in phosphate buffer at pH 7 and 50 mmol L^−1^ in KCl, reading the current peak at −0.58 V. Comparing this curve with the analogous “blank” curve built on the first day in the absence of proline in solution [see “blank” curve, i.e., curve (d) in Figure 3A], a significant increase in the calibration sensitivity is observed. Similar calibration curves were also built in the days following the sensor assembly, recording the sensor response for about a week. Obviously, a fresh solution containing proline was prepared each day, and the sensor was rinsed with water and stored in air at the end of measurement period. The calibration curves on the third and seventh day are also reported in Figure 3A [curves (b) and (c), respectively]. Figure 3B shows the corresponding calibration straight-lines of curves in Figure 3A, with the relative confidence intervals, and in Figure S5A,B, the bar-charts of the slope and linearity range values are displayed, respectively.

Finally, in Table 1 the equation of the straight line constructed using CV on the first day and that of the blank curve are summarized.

**Figure 4:**
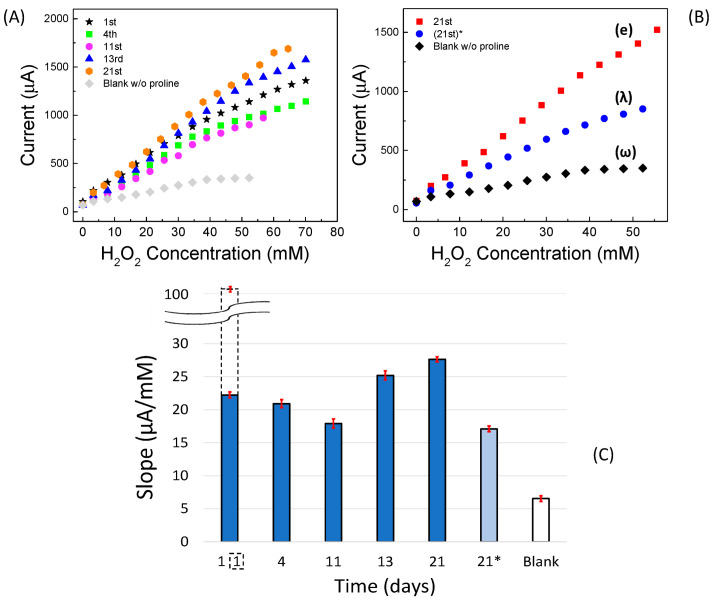
Non-enzymatic catalytic (GC-Ag_paste_)-proline-assisted LDH amperometric sensor. (**A**) Calibration curves as a function of lifetime including the “blank” curve recorded on the first day. (**B**) Comparison among calibration curves obtained on the (e) 21st day, (λ) 21st day without proline addition in solution, using the same sensor, and (ω) blank, recorded on the first day. Each point is the mean of at least three determinations. (**C**) Bar chart of straight-line slopes as a function of lifetime. Dotted bar indicates the slope of the calibration curve recorded on the first day under nitrogen stream, whereas 21* (i.e., the slope of λ curve) is that on the 21st day without new addition proline in solution.

Since the CVs (see Figure 2A) clearly showed a positive effect due to the presence of proline, we also tested its effect on amperometric measurements vs. H_2_O_2_. In this case, as previously specified, the measurements were performed under stirring. A schematic representation of the experimental setup is displayed in Figure S6A. Several of the measured amperometric calibration curves, recorded on the first day up to approximately three weeks and including the “blank” curve, are reported in Figure 4A. The working electrode was biased at −0.58 V vs. the reference electrode and dipped in the PBS containing proline (20 mmol L^−1^). Respective straight-lines and their confidence intervals are shown in Figure S7A–E. The straight-line relative to the blank curve without proline in PBS recorded on the first day (ω) and the calibration straight-line (λ) recorded on 21st day using a fresh buffer solution, but without any new addition of proline, are also shown in Figure 4B.

Interestingly, it can be seen that after three weeks of repeated use, during which the catalytic sensor was regularly polarized at −0.58 V and immersed in the freshly prepared 20 mmol L^−1^ solution of proline each time, the fabricated sensor still provided a good catalytic response towards H_2_O_2_. However, it is interesting to note that a not-negligible response was also achieved even if the measurement was carried out without the usual addition of proline in solution, i.e., with the same sensor used in previous measurements immersed only in phosphate buffer and KCl solution.

These results can be explained by looking to the values of the straight-line slopes reported in Figure 4C. Indeed, up to about the 11th day from the preparation, the slope tends to decrease slowly, probably because the modest organic part of the Ag_paste_, weakly contained on the pristine silver paste, solubilizes or disperses in solution, and therefore, some small granules of LDH tend to detach from the sensor, while the layer of the silver paste, originally opaque, becomes more and more spongy and brighter. Afterwards, the slope tends to increase, at least until the 21st day of use, reaching a value even higher than that of the 1st day. This happens because, to this point, the sensor is affected both by the concentration of proline added to solution each time and by the proline which remains attached to the sensor after each amperometric experiment, continuing to interact with the remaining LDH, that no longer detaches from the silver paste. As a matter of fact, it is widely reported in the literature that many amino acids can interact very well with various types of LDH [45,53], to the point of being permanently immobilized on them. It was also shown [37] that by carrying out cyclic voltammetry in solutions containing proline and AgNO_3_, the proline attached to nanoparticles or other surfaces made of silver, probably by electro-polymerization. As far as our experiments are concerned, by repeatedly immersing our sensor, biased at −0.58 V, in a PBS and KCl solution containing 20 mmol L^−1^ proline under magnetic stirring, a non-negligible amount of proline remained reasonably fixed on the sensor, which in fact acted as a good catalyst towards hydrogen peroxide even without further additions of free proline in solution.

The possibility of interference for our catalytic (GC-Ag_paste_)-proline-assisted LDH amperometric sensor was investigated by studying its selectivity in the presence of different antioxidant substances and of some highly oxidizing species present in solution. The results were compared with the selectivity observed for the enzymatic and non-enzymatic sensors of the same type, but not proline assisted, previously studied by us [34], as summarized in Table 2. The proline-assisted LDH sensor does not suffer from any interference from the different antioxidant substances studied, such as glucose, uric acid, folic acid, nitrite ion, and even by ascorbic acid, while it naturally suffers from the presence of strong oxidants such as nitrate, Fe (III), or permanganate, in the same way or, in some cases, even slightly less (see the case of nitrate), compared to previous non-proline-assisted sensors [34].

Finally, as already mentioned, the most interesting determinations of hydrogen peroxide are those achievable in real samples containing very small concentrations of H_2_O_2_, so we wondered if it was possible to further increase the sensitivity of our proline-assisted LDH catalytic sensor and, at the same time, also decrease the value of its limit of detection. In this regard, purging the solution with an inert gas such as nitrogen or argon was demonstrated to be beneficial as it reduced the interference of the oxygen contained in air when sensing the oxygen resulting from hydrogen peroxide oxidation reaction [54,55,56]. Indeed, under deoxygenated conditions, the lowest detectable signal is that effectively due to the reduction of oxygen, only resulting from the catalytic oxidation of H_2_O_2_ (see Reactions (1) and (2) reported above in the Introduction section) and not by the reduction of oxygen coming from the air and dissolved in solution. We therefore decided to repeat the construction of a calibration curve with our (GC-Ag_paste_)-proline-assisted LDH sensor, using a sealed thermostated cell under a nitrogen stream as schematized in Figure S6B.

**Figure 5:**
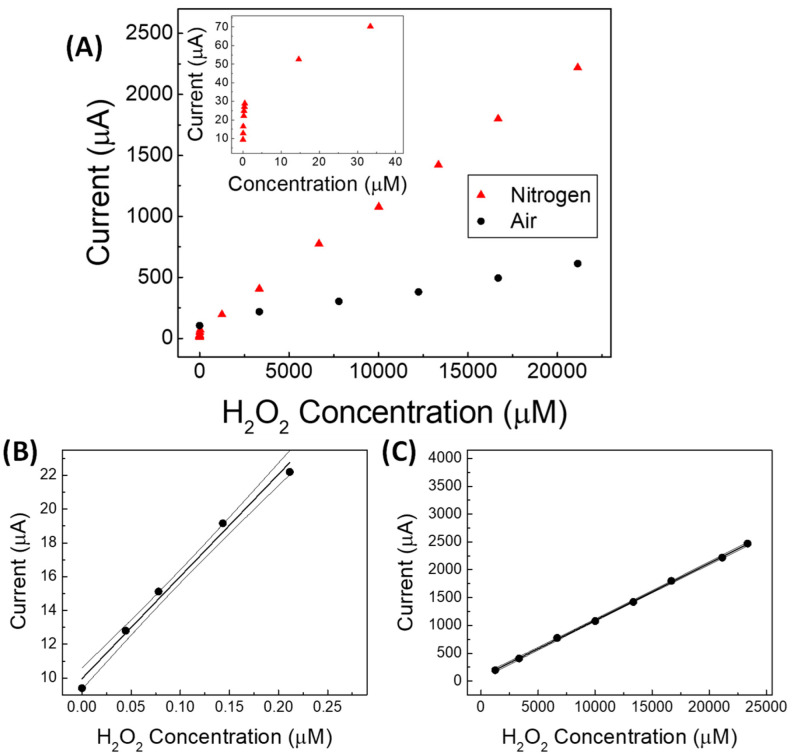
(**A**) Amperometric calibration curves of non-enzymatic (GC-Ag_paste_)-catalytic proline-assisted LDH sensor under static air and under N_2_ stream from 0.05 to 20,000 µmol L^−1^ of hydrogen peroxide; in the inset from about 0.05 to 40 µmol L^−1^. (**B**,**C**) straight lines and confidence intervals at low and at high concentration ranges of hydrogen peroxide, respectively, for the proline-assisted LDH sensor operated under N_2_ stream. Current straight lines obtained by subsequent additions of 150–200 µL of 3 × 10^−5^ % H_2_O_2_ water solution (**B**) and of 3 % H_2_O_2_ water solution (**C**).

In Figure S8, a raw amperometric calibration curve obtained in PBS solution containing proline, under static air (with 21% of oxygen) (Figure S8A), is compared with that obtained by operating in the same solution deoxygenated by N_2_ (Figure S8B). The two respective calibration curves are displayed in Figure 5A. A significant difference in the calibration sensitivity between the two operating conditions can be clearly observed, as previously shown in Figure 4C, where the slope values of the two straight lines obtained at first day of the lifetime of our sensor operated in air (black bar 1) or under nitrogen (white dotted bar, framed 1) are reported, respectively.

Interestingly, as shown in the inset of Figure 5A, two linearity ranges (Figure 5B,C) were found by the sensor operating in deoxygenated solution, the first one being at very low concentrations (0.05 to 0.22 µmol L^−1^), and the second one at higher concentrations (0.0012 to 23.3 mmol L^−1^). This behavior in which the linearity of the response to H_2_O_2_ is generally divided into at least two different ranges is well known when operating with catalytic sensors for H_2_O_2_ under a nitrogen stream [37,38].

In Table 3, straight-lines equations and best analytical data (with analytical method validation) and data comparison between the present work and from our previous study [34] are summarized. For comparison, data from some of the other main studies concerning catalytic sensors for H_2_O_2_ recently reported on in the literature and found by other authors are also tabulated in Table S3 [37,38,57,58,59,60] in the Supplementary Materials.

**Figure 6:**
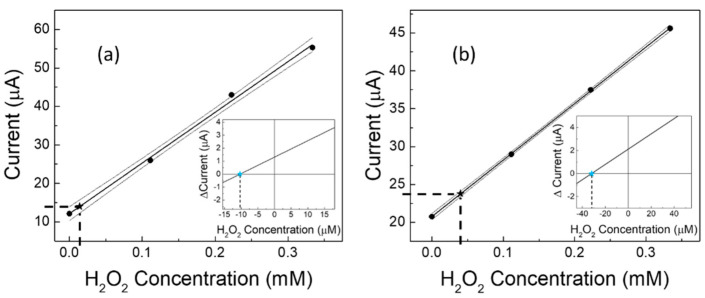
Measurement of H_2_O_2_ concentration in urine samples from (**a**) healthy and (**b**) diabetic subjects. Experimental calibration curves and confidence intervals are built in matrix and hydrogen peroxide concentrations are found by using the linear regression method and Gran’s plot method (in the insets). Each point (black filled circles) represents the mean of at least three repeated determinations. Black star symbols and dotted lines represent the experimental application of the regression equation method. In the insets, coloured star symbols represent the experimental application of the Gran’s plot method.

Given the improved performance under a nitrogen stream, it was thus possible to use our catalytic (GC-Ag_paste_)-proline-assisted LDH sensor for a difficult application in real samples [37,61,62], i.e., the determination of the level of H_2_O_2_ present in human urine, both from a healthy subject and from a diabetic subject. Due to the very low concentrations of hydrogen peroxide contained in these biological matrices, the determinations were performed by constructing calibration curves “in matrix”, then calculating the contained H_2_O_2_ values, applying both the regression equation method [37] and the Gran’s plot method [49,50]. The results are shown in Figure 6a,b for the healthy and the diabetic subjects, respectively. The calculations by Gran’s plot method are displayed in the insets. The obtained data are also summarized in Table 4, which includes results from three works reported in the literature [37,61,62] for similar samples from human subjects with the two different health conditions. It is worth noting that, as expected, the level of H_2_O_2_ in the diabetic subject is always greater than (about double) that found in the healthy subject.

Looking to the values reported in Table 4, it is also possible to evaluate the accuracy of the proposed method by comparing our hydrogen peroxide data with the values reported in the literature relating to healthy or diabetic patients. It can be observed that the order of magnitude of the values found by us it is practically coincident with that of the data available in the literature.

A further estimation of the accuracy of the method was achieved by applying the “standard addition” method “in matrix”, even though, as is known, the application of this method provides information that is necessary but not entirely sufficient to establish the accuracy of a method (see Table 5). From the results it can regardless be observed that the percentage recoveries were always between 95.1% and 99.8%; therefore the percentage recoveries are certainly satisfactory.

Based on our findings, the presence of proline clearly improves the response of the catalytic sensor. Naturally, we wondered what the mechanism was thanks to which the proline improves the response of the sensor, both by significantly increasing the calibration sensitivity and by lowering the LOD value. In this regard, the literature does not report very concordant opinions and is therefore not entirely convincing. Further difficulties in interpreting the different points of view of different authors derive from the fact that some have developed their methods based on the voltammetric oxidation peaks of H_2_O_2_ and others on the reduction of oxygen. The only authors who dwell long enough on the subject are F. Zhao et al. [38], who built a GC sensor on which Ag nanoparticles coated with proline were electrodeposited. According to these authors, the role of proline would, in practice, simply be that of “slowing” the oxidation of Ag^0^ and the “superior properties” of their sensor should be attributed above all to the stabilizing effect of L-proline which disadvantages the reoxidation of the metallic silver to the silver oxide, slowing the formation of silver oxides, as well as assisting the formation of silver nanoparticles highly dispersed on the GC surface. Since these authors do not use any type of LDH in the construction of their sensor, they therefore attribute all of the positive effects of proline to the interaction between it and the surface of the silver nanoparticles. Additionally, M.L. Charitra et al. [39], who proposed a catalytic sensor for estriol, claim that polyproline would modify the response of their CP transducer, enhancing the electrochemical oxidation of the estriol. Instead, L. Xu et al. [40], who, for the operation of their H_2_O_2_ sensor do not use proline but gold nanoparticles on a transducer modified with LDH containing cobalt and manganese ions, observe a “catalytic current” relative to the voltammetric “oxidation” peak of H_2_O_2_ much higher than in the case in which the same transducer was not modified by LDH. M. Asif et al. [37], who used LDH containing copper and aluminium ions, in contact with nanospheres of iron oxides, to build their sensor for H_2_O_2_—that is, without the use of proline—nevertheless managed to reach an extraordinary LOD value of the order of nmol L^−1^ and highlight the benefits of using LDH. H. Heli et al. [36], who do not use proline for their H_2_O_2_ sensor based on a modified CP transducer with multiwall carbon nanotubes in contact with LDH-based cobalt and aluminium, argue that LDH catalyses both the electrooxidation of hydrogen peroxide and the electroreduction process. Therefore, according to these authors, it is LDH above all that exerts highly positive action, strongly increasing the catalytic current. In the light of these quite different points of view, we have also tried to understand whether the effect of proline, which, as experimentally found, significantly increases the sensitivity of our catalytic amperometric and voltammetric sensors, consists of only a positive cleaning effect that stabilizes metallic silver, or whether there are other effects through which it intervenes, for example, facilitating the mechanisms of electron transfer [37], typical of the metal ions contained in LDH.

**Figure 7:**
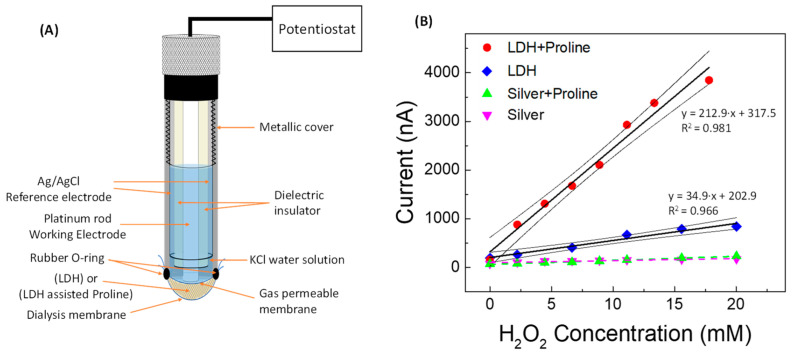
(**A**) Schematic of the non-enzymatic catalytic Clark-type proline-assisted (or non-assisted) LDH amperometric sensor and (**B**) straight line calibration curve vs. H_2_O_2_ concentration obtained using Clark sensor with (●) proline-assisted LDH, (◆) LDH without proline, (▲) proline-assisted silver paste, and (▼) silver paste without proline.

To obtain some experimental feedback in this regard, we fabricated a Clark-type gaseous diffusion amperometric electrode, whose schematic diagram is shown in Figure 7A, very similar to the one we built in a previous work [35], but of course non-enzymatic.

In order to separately investigate the role of proline, LDH, and silver paste on the overall catalytic response, calibration curves for H_2_O_2_ were built with the transducer being modified by placing between the gas-permeable membrane and the dialysis membrane a mixture of LDH (Zn-Al-NO_3_) and proline (the same quantity contained in 20 mmol L^−1^ of PBS solution), or, alternatively, LDH alone or silver paste and proline, or silver paste alone, always operating in PBS solution. In Figure 7B it can be observed that the constructed amperometric straight line obtained using LDH assisted by proline has a much higher slope value than that obtained by operating using LDH without proline. Proline therefore significantly increases the sensitivity of the LDH-based sensor towards H_2_O_2_, even without the addition of silver paste, so that any cleaning effect of the Ag^0^ cannot occur. On the other hand, the silver alone, coming from the silver paste, does not exert by itself a consistent catalytic activity, and the effect of proline addition to the silver paste is almost negligible, showing both these calibration curves to have a very low slope. All this leads to the conclusion that, at least in our case, LDH is essential to improve the performance of the sensor and that proline plays a highly positive role in the presence of LDH, probably facilitating the electronic transfer processes through the metal ions contained in the LDH.

## 4. Conclusions

In the present research, we demonstrated that by operating with a non-enzymatic (GC-Ag_paste_)-LDH catalytic amperometric sensor with proline assistance the calibration sensitivity is increased up to about 30 times. The presence of proline clearly increased the catalytic activity of LDH rather than that of the silver coming from the silver paste spread on the GC transducer. It was also observed that, by operating under a nitrogen atmosphere instead of under static air, the LOD of the method can be significantly lowered, from 0.15 mmol L^−1^ to 0.05 µmol L^−1^. Furthermore, we observed that the sensor was affected only by the possible interference of very strong oxidizing species, rarely present in real samples, while it is not affected at all by the most common antioxidant substances found in real samples. Lastly, the fabricated (GC-Ag_paste_) proline-assisted LDH catalytic amperometric sensor, thanks to the great sensitivity towards hydrogen peroxide and to the very low sensitivity to interfering species, could be advantageously used for the analysis of H_2_O_2_ in real samples, for instance biological fluids such as human urine samples, since it was capable of distinguishing between samples coming from healthy subjects from those belonging to diabetic patients. This sensor is therefore an effective alternative to similar enzymatic biosensors, more delicate and complex, but ultimately certainly more expensive.

## Figures and Tables

**Table 1:** CV method validation: main analytical data and straight-line equations of the calibration curves at the 1st day and blank curve.

	Linear Regression (y = mA; x = mM)	Linearity Range (mM)	R^2^	LOD (mM)	RSD %
1st day	y = (0.256 ± 0.0130)∙x + (9.819 ± 0.1110)	0–17	0.9899	0.5	2.25
blank	y = (0.0676 ± 0.00460)∙x + (8.597 ± 0.0680)	0–30	0.9822	0.5	2.25

**Table 2:** Interferences in hydrogen peroxide determination expressed as the percentage response variation to H_2_O_2_ using the non-enzymatic catalytic (GC-Ag_paste_)-proline-assisted LDH amperometric sensor and comparison with those observed in the previously developed enzymatic or simply catalytic (GC-Ag_paste_)-LDH sensors [34]. Each added interfering species had a final concentration in solution equal to that of hydrogen peroxide.

	Non Enzymatic Catalytic (GC-Ag_paste_)-Proline Assisted LDH Sensor	Enzymatic or Catalytic (GC-Ag_paste_)-LDH Sensors (Not Proline Assisted) [34]
Glucose	0%	-
Uric acid	0%	-
Ascorbic acid	≈0%	−5.6%
Sodium nitrite	0%	−1%
Sodium nitrate	+1%	+3%
Fe^3+^	≈+30%	+32%
Potassium permanganate	+440%	+1300%

**Table 3:** Analytical method validation. Straight-line equations and best analytical data of the sensor studied in the present paper and in our previous study [34].

Best Analytical data of GC-LDH-Catalase Enzymatic biosensor studied in previous work and method validation								
Linear Regression	Linearity Range (mM)	R^2^	LOD (mM)	LOQ (mM)	RSD%	Response time (s)	Lifetime (days)	Ref
y = (10.09 ± 0.29) x + (115.1 ± 29.1)	0.25–158	0.9976	0.2	0.6	0.5	8.5	75	[34]
(y = μA; x = mM)								
Best Analytical data of non-enzymatic catalytic LDH sensor studied in previous work and method validation								
Linear Regression	Linearity Range (mM)	R^2^	LOD (mM)	LOQ (mM)	RSD%	Response time (s)	Lifetime (days)	Ref
y = (0.966*2* ± 0.010*9*) x + (−102.6 ± 8.85)	144.5–1195.2	0.9986	1	3	1.8	17.5	68	[34]
(y = μA; x = mM)								
Best Analytical data of non-enzymatic catalytic proline assisted LDH sensor studied in this work and method validation								
(operating under static air)								
Linear Regression	Linearity Range (mM)	R^2^	LOD (mM)	LOQ (mM)	RSD%	Response time (s)	Lifetime (days)	Ref
y = (27.407 ± 0.450) x + (83.466 ± 8.713)	0.3–33.4	0.9981	0.15	0.3	5	7	≥21	This work
(y = μA; x = mM)								
Best Analytical data of non-enzymatic catalytic proline assisted LDH sensor studied in this work and method validation								
(operating under nitrogen—low concentration range)								
Linear Regression	Linearity Range (mM)	R^2^	LOD (mM)	LOQ (mM)	RSD%	Response time (s)	Lifetime (days)	Ref
y = (30874.1 ± 3685.7) x + (15.12 ± 1.17)	0.00005–0.00022	0.9561	0.00005	0.0001	5	10	>3	This work
(y = μA; x = mM)	0.05–0.22 (µM)		0.05 (µM)	0.1 (µM)				
Best Analytical data of non-enzymatic catalytic proline assisted LDH sensor studied in this work and method validation								
(operating under nitrogen—high concentration range)								
Linear Regression	Linearity Range (mM)	R^2^	LOD (mM)	LOQ (mM)	RSD%	Response time (s)	Lifetime (days)	Ref
y = (102.9 ± 1.2) x + (66.9 ± 16.3)	0.012–23.3	0.9995	0.005	0.01	2.5	10	>3	This work
(y = μA; x = mM)								

**Table 4:** Experimental values of hydrogen peroxide concentration found in fresh human urine from healthy and diabetic subjects, determined in this work under nitrogen atmosphere, comparing them with those present in three works reported in the literature.

	Exp. Values [µM] Found by Regression Equation Method (This Work)	Exp. Values [µM] Found by Gran’s plot Method (This Work)	Exp. Values [µM] Reported in Literature [61,62]	Exp. Values [µM] Reported in Literature [37]
Healthy subject	30.0 ± 1.00	21.0 ± 1.30	20 ± 1.4	35.4
Diabetic subject	80.0 ± 4.00	64.0 ± 4.20	42 ± 0.72	“higher than healthy”

**Table 5:** Experimental percentage recovery for hydrogen peroxide addition in healthy and diabetic urine samples. The reported found and added concentration values are the final ones obtained after dilution of the solution.

Sample	Found Concentration in Urine Sample (µM)	H_2_O_2_ Additions (µM)	Found + Added Nominal Value (µM)	Experimental Value (µM)	Δ (%) (RSD% = 0.7)	Percent Recovery (RSD% = 0.7)
Healthy	15.0	96.0	111.0	105.6	−4.9	95.1
Healthy	15.0	318.0	333.0	328.0	−1.5	98.5
Diabetic	40.0	71.0	111.0	110.2	−0.7	99.3
Diabetic	40.0	293.5	333.5	332.8	−0.2	99.8

## Supplementary Materials

The following supporting information can be downloaded at: https://www.mdpi.com/article/10.3390/s22197159/s1, Figure S1: LDH structure that we used in this research; Figure S2: X-ray diffraction patterns of LDH grown by the coprecipitation method and LDH grown by hydrothermal method; Figure S3: Comparison of our (GC-Ag_paste_)-LDH non enzymatic sensor previously built and the new (GC-Ag_paste_)-proline assisted LDH non enzymatic sensor; Figure S4: Optimization of proline concentration; Figure S5: Slope and linearity range as a function of lifetime; Figure S6: Three electrodes amperometric systems working in static air and under nitrogen stream; Figure S7: Straight-lines and confidence intervals as a function of lifetime of the non-enzymatic catalytic (GC-Ag_paste_)-proline assisted LDH amperometric sensor; Figure S8: Raw amperometric calibration curves of non-enzymatic catalytic (GC-Ag_paste_)-proline assisted LDH sensor in static air and under nitrogen stream; Table S3: Analytical results from some main recent works, reported in literature, concerning sensors of the same type.
