# Supplementary material for: Novel Electrochemical Sensors Based on L-Proline Assisted LDH for H2O2 Determination in Healthy and Diabetic Urine
Source: Sensors (Basel). 2022 Sep 21;22(19):7159. doi: 10.3390/s22197159 (PMC9572033; doi:10.3390/s22197159)
Supplement: Supplementary file 1 [file sensors-22-07159-s001.zip › sensors-1850729-supplementary.pdf]

Supplementary Materials

Novel Electrochemical Sensors Based on L-Proline Assisted LDH for H_2O_2 Determination in Healthy and Diabetic Urine

Mauro Tomassetti [1,2,*], Riccardo Pezzilli [3,*], Giuseppe Prestopino [3], Corrado Di Natale [1], Pier Gianni Medaglia [3]

[1] *Department of Electronic Engineering, University of Rome ''Tor Vergata'', Viale del Politecnico 1, 00133 Rome, Italy*

[2] *Department of Chemistry, University of Rome, 'La Sapienza", P.le A. Moro 5, 00185 Rome, Italy*

[3] *Department of Industrial Engineering, University of Rome ''Tor Vergata'', Viale del Politecnico 1, 00133 Rome, Italy*

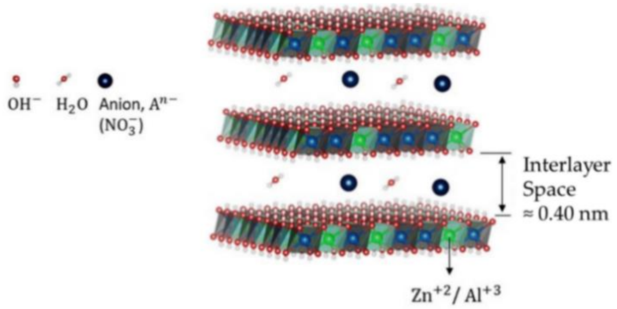

Figure S1. LDH structure that we used in this research.

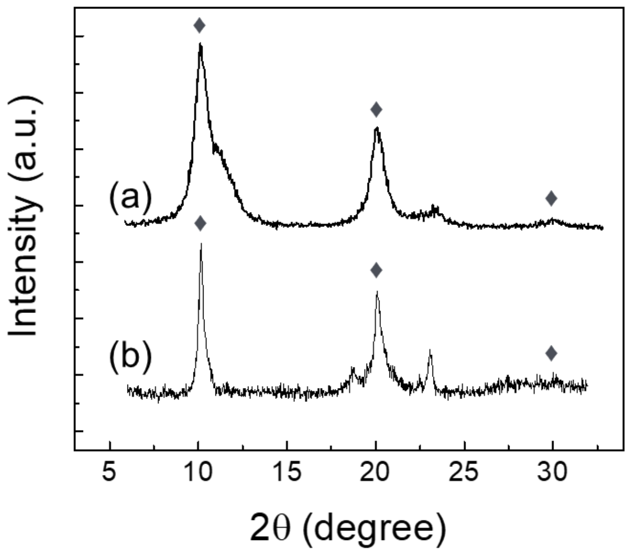

Figure S2. X-ray diffraction patterns of (a) LDH grown by the coprecipitation method, and (b) LDH grown by hydrothermal method. The main basal reflections of the (Zn–Al–NO_3) LDH phase are labelled by diamonds (♦).

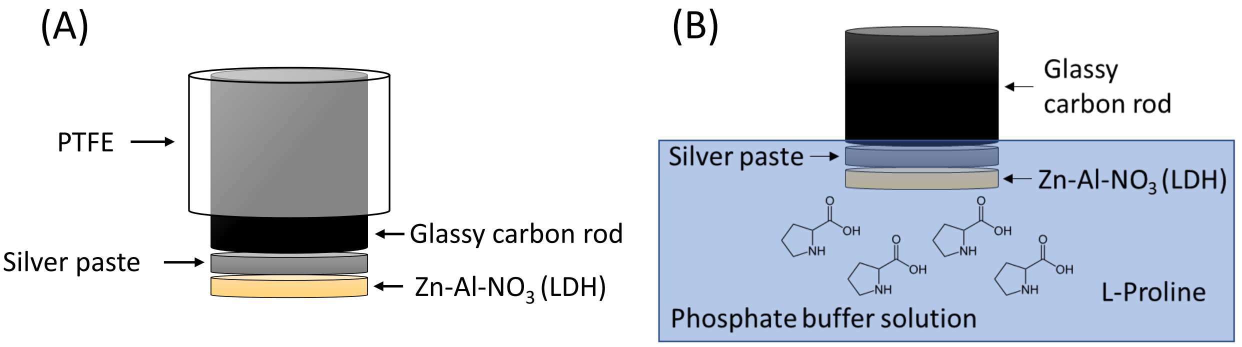

Figure S3. Comparison of our (A) (GC-Ag_{paste})-LDH non enzymatic sensor previously built and (B) new (GC-Ag_{paste})-proline assisted LDH non enzymatic sensor.

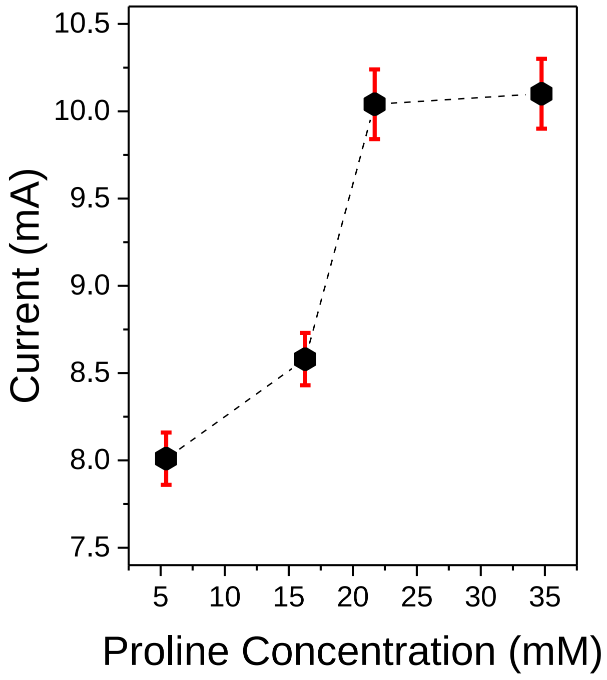

Figure S4. Optimization of proline concentration in the phosphate buffer solution, containing 3.34 mmol L^{-1} of H_2O_2, each point is the mean of at least three determinations.

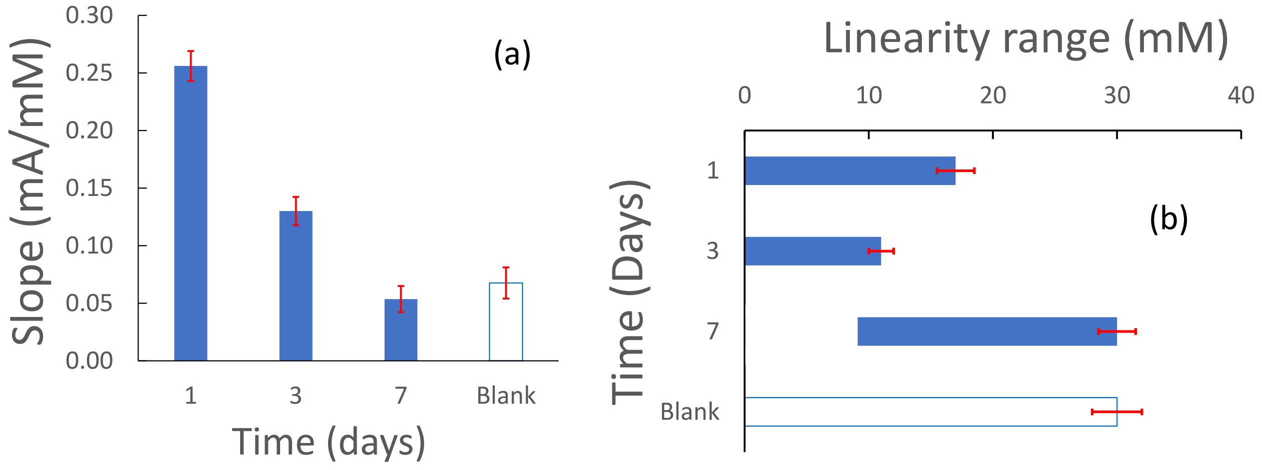

Figure S5. (a) Slope and (b) linearity range values, as bar chart, as a function of lifetime, including blank values recorded on the first day.

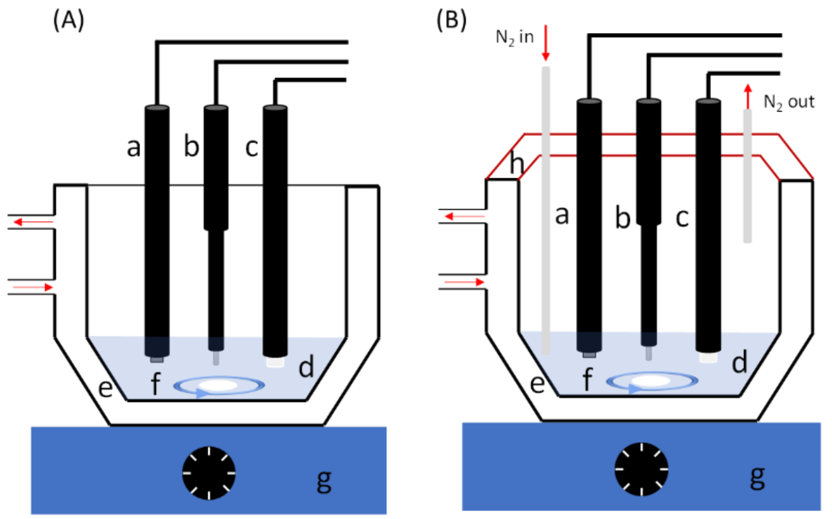

Figure S6. Three electrodes amperometric system used in the present research: (a) working electrode, (b) reference electrode, (c) counter electrode, (d) proline in PBS, (e) thermostated electrochemical cell, (f) magnetic fly, (g) magnetic stirrer, (h) airtight lid. (A) measurement system working in static air and (B) measurement system working under nitrogen stream.

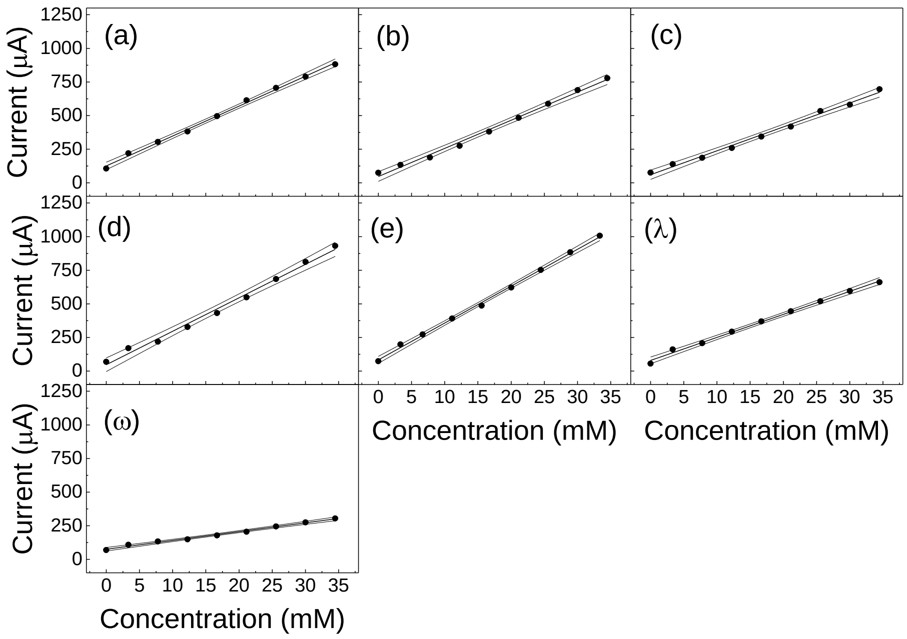

Figure S7. Non-enzymatic catalytic (GC-Ag$_{paste}$)-proline assisted LDH amperometric sensor. Straight-lines and confidence intervals as a function of lifetime: (a) 1st day, (b) 4th day, (c) 11st day, (d) 13rd day, (e) 21st day, (λ) 21st day without new addition of proline in solution, and (ω) blank without proline in PBS solution recorded on the first day.

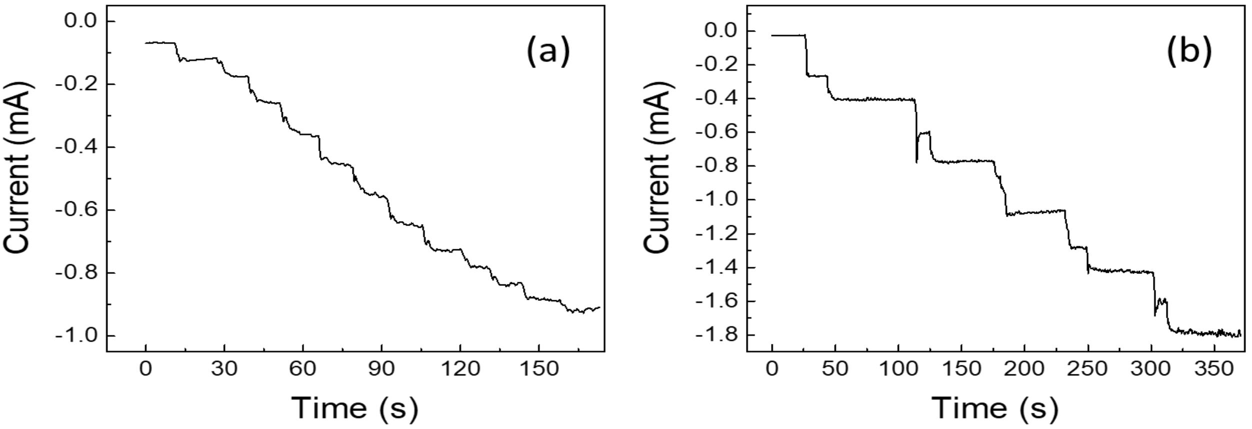

Figure S8. Raw amperometric calibration curves of non-enzymatic catalytic (GC-Ag_{paste})-proline assisted LDH sensor, performed under stirring: (a) in static air and (b) under nitrogen stream. Current variation follows subsequent additions of 150-200 μL of 3% H_2O_2 water solution.

Table S3. Analytical results from some main recent works, reported in literature, by other authors, concerning the same topics, i.e., sensors for hydrogen peroxide.

Best Analytical data of non-enzymatic catalytic proline assisted Ag° sensor reported in the literature (low concentration range)								
Linear Regression	Linearity Range (mM)	R^2	LOD (mM)	LOQ (mM)	RSD%	Response time (s)	Lifetime (days)	Ref
y = (1138) x + (0.319) (y=μA; x=mM)	0.0001-1.918 0.1-1918 (μM)	0.994	0.00005 0.05 (μM)				30	[1]
**Best Analytical data of non-enzymatic catalytic proline assisted Ag° sensor reported in the literature (high concentration range)**								
Linear Regression	Linearity Range (mM)	R^2	LOD (mM)	LOQ (mM)	RSD%	Response time (s)	Lifetime (days)	Ref
y = (860) x + (0.925) (y=μA; x=mM)	1.918-5.145	0.993					25	[1]
**Best Analytical data of non-enzymatic catalytic iron oxide-LDH sensor reported in the literature (low concentration range)**								
Linear Regression	Linearity Range (mM)	R^2	LOD (mM)	LOQ (mM)	RSD%	Response time (s)	Lifetime (days)	Ref
---	0.000003-1.05 0.003-1050 (μM)	0.995	0.000001 0.001 (μM)				25	[2]
**Best Analytical data of non-enzymatic catalytic iron oxide-LDH sensor reported in the literature: (high concentration range)**								
Linear Regression	Linearity Range (mM)	R^2	LOD (mM)	LOQ (mM)	RSD%	Response time (s)	Lifetime (days)	Ref
---	1.5-10.1	0.997					25	[2]
**Best Analytical data of non-enzymatic catalytic Ag-LDH sensor reported in the literature (operated under nitrogen)**								
Linear Regression	Linearity Range (mM)	R^2	LOD (mM)	LOQ (mM)	RSD%	Response time (s)	Lifetime (days)	Ref
---	0.01-19.33	0.9991	0.0022			3		[3]

Best Analytical data of non-enzymatic catalytic Ag-ZnO sensor reported in the literature

Linear Regression	Linearity Range (mM)	R^2	LOD (mM)	LOQ (mM)	RSD%	Response time (s)	Lifetime (days)	Ref
y = (0.1156) x + (23.96) (y=A; x=M)	0.002-5.5	0.9984	0.42 (μM)			10		[4]

Best Analytical data of non-enzymatic catalytic Ag-Ppy-Fe3O4 sensor reported in the literature (operated under nitrogen)

Linear Regression	Linearity Range (mM)	R^2	LOD (mM)	LOQ (mM)	RSD%	Response time (s)	Lifetime (days)	Ref
y = (1.310) x + (0.3183) (y=μA; x=mM)	0.005-11.55	0.9976	1.7 (μM)			5		[5]

Best Analytical data of non-enzymatic catalytic Ag-CQDs sensor reported in the literature (operated under nitrogen)

Linear Regression	Linearity Range (mM)	R^2	LOD (mM)	LOQ (mM)	RSD%	Response time (s)	Lifetime (days)	Ref
y = (-1.614) x + (-10.67) (y=μA; x=μM)	0.2-27.0 (μM)	0.9937	0.08 (μM)			2		[6]

Supplementary References

1. Zhao, F.; Zhou, M.; Wang, L.; Huang, Z.; Chu, Y. One-Step Voltammetric Deposition of l-Proline Assisted Silver Nanoparticles Modified Glassy Carbon Electrode for Electrochemical Detection of Hydrogen Peroxide. *Journal of Electroanalytical Chemistry* **2019**, *833*, 205–212, doi:10.1016/j.jelechem.2018.11.050.
2. Asif, M.; Liu, H.; Aziz, A.; Wang, H.; Wang, Z.; Ajmal, M.; Xiao, F.; Liu, H. Core-Shell Iron Oxide-Layered Double Hydroxide: High Electrochemical Sensing Performance of H_2O_2 Biomarker in Live Cancer Cells with Plasma Therapeutics. *Biosensors and Bioelectronics* **2017**, *97*, 352–359, doi:10.1016/j.bios.2017.05.057.
3. Yang, Z.; Tjiu, W.W.; Fan, W.; Liu, T. Electrodepositing Ag Nanodendrites on Layered Double Hydroxides Modified Glassy Carbon Electrode: Novel Hierarchical Structure for Hydrogen Peroxide Detection. *Electrochimica Acta* **2013**, *90*, 400–407, doi:10.1016/j.electacta.2012.12.038.
4. Wang, Q.; Zheng, J. Electrodeposition of Silver Nanoparticles on a Zinc Oxide Film: Improvement of Amperometric Sensing Sensitivity and Stability for Hydrogen Peroxide Determination. *Microchim Acta* **2010**, *169*, 361–365, doi:10.1007/s00604-010-0356-7.
5. Qi, C.; Zheng, J. Novel Nonenzymatic Hydrogen Peroxide Sensor Based on Fe3O4/PPy/Ag Nanocomposites. *Journal of Electroanalytical Chemistry* **2015**, *747*, 53–58, doi:10.1016/j.jelechem.2015.04.004.
6. Jahanbakhshi, M.; Habibi, B. A Novel and Facile Synthesis of Carbon Quantum Dots via Salep Hydrothermal Treatment as the Silver Nanoparticles Support: Application to Electroanalytical Determination of H2O2 in Fetal Bovine Serum. *Biosensors and Bioelectronics* **2016**, *81*, 143–150, doi:10.1016/j.bios.2016.02.064.
